# Acute Hemiballismus as the Initial Manifestation of Ischemic Stroke: A Case Report

**DOI:** 10.5811/cpcem.2021.5.52678

**Published:** 2021-07-28

**Authors:** Huiling Huang, Siang-Hiong Goh

**Affiliations:** Changi General Hospital, Department of Accident and Emergency, Singapore

**Keywords:** Case report, hemiballismus, hemichorea, cerebrovascular disease

## Abstract

**Introduction:**

Cerebrovascular disease often presents with “negative” symptoms such as weakness with reduced movement of body parts or sensory loss. Rarely do “positive” symptoms such as abnormal movements manifest in acute stroke, with hemichorea being a very rare manifestation.

**Case Report:**

This is a case report of a 62-year-old chronic smoker with no known past medical history who presented with choreatic movements of his arm and leg. Magnetic resonance imaging of the brain showed changes consistent with an infarct in the right centrum semiovale. He was treated with dual antiplatelets and was noted to have subsequent improvement in symptoms.

**Conclusion:**

Recognition and awareness of stroke presenting as movement disorders in the emergency department can help prevent delays in diagnosis and treatment.

## INTRODUCTION

Hemichorea-hemiballismus (HCHB) is a relatively rare hyperkinetic movement disorder characterized by involuntary, coarse, and wide-amplitude movements involving the unilateral arm and leg. It is a rare manifestation of stroke and is reported to be most commonly due to contralateral lesions in the subthalamic nucleus and basal ganglia.^1^,^2^ In recent years, however, there have been reports of cortical strokes presenting with HCHB.^4^,^5^ Based on previous literature, the incidence of HCHB in acute ischemic stroke ranges between 0.4%–0.54%.^1^,^3^

We report a case of acute onset hemiballismus as the initial manifestation of acute infarct in the centrum semiovale, which improved after treatment with antithrombotic therapy.

## CASE REPORT

A 62-year-old Chinese male, chronic smoker of 40 pack-years with nil past medical history, presented to our emergency department (ED) with intermittent episodes of abnormal involuntary movement of his left arm and leg for three days. He described them as swinging movements of the left arm at the wrist and elbow joint that could be suppressed with the other hand. There was also associated numbness of the left hand and unsteady gait with gait deviation to the left on ambulation. He had no involuntary movement of the face and no complaint of slurred speech. He also denied any use of long-term medications including illicit substances or traditional medications. There was no family history of any neurological disease.

General examination revealed no abnormal finding. On neurological examination, he was noted to have hemiballismus mixed with chorea-like movements over the left upper and lower limbs. There was no pronator drift, and power and sensation was full over bilateral upper and lower limbs. Cranial nerve exam, cerebellar examination was normal. He was noted to be normoglycemic as well. An urgent computed tomography of the brain done in the ED was reported by the radiologist to have no evidence of acute intracranial haemorrhage, acute territorial infarct, or mass effect. The electrocardiogram done in the ED revealed sinus rhythm.

The patient was admitted to the medical general ward for further workup for his hemiballismus-chorea. Initial tests performed included iron studies, ceruloplasmin levels, an infective screen (hepatitis B/C/human immunodeficiency virus/syphilis), calcium, magnesium, and phosphate. All were reported normal. The following day the patient was seen by a neurologist who arranged for a magnetic resonance imaging (MRI) brain stroke protocol. The MRI of the brain showed a small focus (3 millimeters) of restricted diffusion in the right centrum semiovale (Image). No haemorrhagic conversion or significant perifocal oedema, no midline shift, or hydrocephalus was noted. Magnetic resonance angiogram showed no evidence of flow-limiting stenosis of the arteries of the anterior and posterior circulation. No aneurysm was noted.

He was started on dual anti-platelet therapy for the stroke and atorvastatin for newly diagnosed hyperlipidaemia. Further tests included a two-dimensional echocardiography; 24-hour Holter and hemoglobin A1c were also done for this patient, which were all noted to be normal. Carotid Doppler investigations revealed minor plaques. Prior to discharge, the patient was reviewed by the physiotherapist and occupational therapist and was noted to have improvement in symptoms with very minimal occasional hemiballismus over his left upper limb that did not affect his gait and basic activities of daily living. The patient was discharged after three days of inpatient management.

### Outcome and Follow-up

The patient was reviewed at the neurology clinic three months post discharge. He was noted to have resolution of symptoms and was able to return to work as a bartender. He was then discharged from neurology.

## DISCUSSION

Chorea is a hyperkinetic movement disorder characterized by rapid and unpredictable contractions affecting mostly distal limbs, but also the face and trunk. The movements are involuntary and non-patterned with variable speed, timing, and direction, flowing from one body part to another.^2^ Ballism refers to involuntary movements that are proximal and large in amplitude with a flinging or kicking character. Ballism is most often unilateral (hemiballismus), and although present at rest it becomes more prominent with action.^6^ Often hemichorea and hemiballismus coexist.^7^

Vascular and structural pathologies within the contralateral subthalamic nucleus and basal ganglia are the most common causes of hemiballismus, although lesions in the striatum, thalamus, cerebral cortex, subcortical area, and midbrain are also reported to cause hemiballismus.^7^ In a recent case series, four patients with cortical stroke presented with hemichorea as the primary presentation.^4^ Although vascular infarcts resulting in HCHB may vary in anatomical location, they are localizable to a common functional network.^8^

Both ischemic and haemorrhagic stroke account for the most common etiology of HCHB. In one study 11 of 21 patients had a stroke etiology, and in another series 18 of 25 patients had either ischemic or haemorrhagic stroke.^9^,^10^ Although stroke is a common etiology of HCHB, the incidence of this movement disorder compared to other manifestations of stroke (weakness) is extremely rare, with a range of 0.4% to 0.54%.^1^,^3^ Other causes of HCHB include infection, neoplasm, traumatic brain injury, nonketotic hyperglycemia, autoimmune disorders, and use of dopaminergic drugs. Our patient’s hemiballismus was caused by an infarction in the right centrum semiovale (Image).

**CPC-EM Capsule**

What do we already know about this clinical entity?

*Strokes are the most common etiology of hemichorea-hemiballismus (HCHB). However, incidence of HCHB as the initial manifestation of acute ischemic stroke is rare*.

What makes this presentation of disease reportable?

*Ischemic strokes present with “negative” symptoms such as weakness or sensory loss. Rarely do “positive” symptoms such as abnormal movements manifest in acute stroke*.

What is the major learning point?

*Clinicians should recognize that an acute onset of HCHB can be a symptom of stroke and should be treated and worked up accordingly*.

How might this improve emergency medicine practice?

*Recognition and awareness of stroke presenting as movement disorders can prevent delays in diagnosis and management*.

The centrum semiovale is the central area of white matter found beneath the cerebral cortex. The white matter, located in each hemisphere between the cerebral cortex and nuclei, consists of cortical projection fibres, association fibres, and cortical fibres. Two cases of centrum semiovale stroke causing hemiballismus were reported in recent literature. The first case involved a concurrent occurrence of non-ketotic hyperglycaemia and a stroke.^11^ The second was a case of repeating acute-onset hemiballismus in a single patient who had hemiballismus after an initial acute infarct in the centrum semiovale, complete resolution of symptoms, and re-presentation to the hospital five months later with hemiballismus over the same limbs, but with multifocal patchy, subcortical-restricted diffusion focus on MRI instead.^12^ Our case differs as our patient was of Chinese descent and had delayed his presentation to the ED until three days after the onset of hemiballismus. He was also younger than the mean age of patients with post-stroke hemiballismus noted in current literature.

Hemiballismus patients require treatment both for the underlying etiology of the movement and for the movements themselves. A majority of vascular hemiballismus have a good prognosis, and most resolve spontaneously. A 2010 observational study of 15 patients with post-stroke hemiballismus reported eight patients (53%) not needing any pharmacological treatment due to rapid resolution, while the remaining seven required pharmacological therapy for the control of hemichorea. However, most patients had symptom resolution within two months.^13^ In cases where ballistic movement is persistent, or affecting function, medical therapy with antidopaminergic drugs (haloperidol) can be considered.^13^,^14^ In the case of our patient, it was determined that he had mild hemiballismus which improved prior to the discharge from the hospital and resolution of symptoms by the time of the outpatient neurology follow-up. Hence, dopamine receptor blocker agents were not considered.

## CONCLUSION

We report a rare case of ischemic stroke presenting with acute onset hemichorea-hemiballismus where there was infarction at the right centrum semiovale. Recognition and awareness of stroke presenting as movement disorders in the emergency department can help prevent delays in diagnosis and treatment.

## Figures and Tables

**Image:**
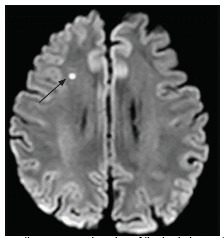
Magnetic resonance imaging of the brain in a patient with hemiballismus demonstrating a small focus (3 millimeters) of restricted diffusion in the right centrum semiovale (arrow).
